# Venous thromboembolism risk score during hospitalization in pregnancy: results of 10694 prospective evaluations in a clinical trial

**DOI:** 10.1016/j.clinsp.2023.100230

**Published:** 2023-06-10

**Authors:** Venina Isabel Poço Viana Leme de Barros, Ana Maria Kondo Igai, Fernanda Spadotto Baptista, Maria Rita de Figueiredo Lemos Bortolotto, Stela Verzinhasse Peres, Rossana Pulcinelli Vieira Francisco

**Affiliations:** Hospital das Clínicas, Universidade de São Paulo (HCUSP), São Paulo, SP, Brazil

**Keywords:** Venous thromboembolism, Hospitals, Pregnancy, Risk factors, Maternal death, Neoplasms

## Abstract

**Objectives:**

Hospitalization during pregnancy and childbirth increases the risk of Venous Thromboembolism Risk (VTE). This study applied a VTE risk score to all hospitalized pregnant women to ascertain its effectiveness in preventing maternal death from VTE until 3 months after discharge.

**Methods:**

In this interventional study, patients were classified as low- or high-risk according to the VTE risk score (Clinics Hospital risk score). High-risk patients (score ≥ 3) were scheduled for pharmacological Thromboprophylaxis (TPX). Interaction analysis of the main risk factors was performed using Odds Ratio (OR) and Poisson regression with robust variance.

**Results:**

The data of 10694 cases (7212 patients) were analyzed; 1626 (15.2%, 1000 patients) and 9068 (84.8%, 6212 patients) cases were classified as high-risk (score ≥ 3) and low-risk (score < 3), respectively. The main risk factors (Odds Ratio, 95% Confidence Interval) for VTE were age ≥ 35 and < 40 years (1.6, 1.4–1.8), parity ≥ 3 (3.5, 3.0–4.0), age ≥ 40 years (4.8, 4.1–5.6), multiple pregnancies (2.1, 1.7–2.5), BMI ≥ 40 kg/m^2^ (5.1, 4.3–6.0), severe infection (4.1, 3.3–5.1), and cancer (12.3, 8.8–17.2). There were 10 cases of VTE: 7/1636 (0.4%) and 3/9068 (0.03%) in the high- and low-risk groups, respectively. No patient died of VTE. The intervention reduced the VTE risk by 87%; the number needed to treat was 3.

**Conclusions:**

This VTE risk score was effective in preventing maternal deaths from VTE, with a low indication for TPX. Maternal age, multiparity, obesity, severe infections, multiple pregnancies, and cancer were the main risk factors for VTE.

## Introduction

Venous Thromboembolism (VTE), comprising deep vein thrombosis and pulmonary embolism, has an annual incidence of approximately 1 per 1000 in adult populations and is a major burden in hospitalized patients. In the United States, nearly 50% of the total estimated annual number of VTE events is related to a current or recent hospitalization. [1] Preventing fatal pulmonary embolism is the primary goal of anticoagulant prophylaxis for VTE. [1] The 1-month case fatality rate for VTE ranges from 2.8% to 12%, and the initial presentation for 24% of patients with pulmonary embolism is sudden death. [2]

The risk of hospital-acquired VTE is reduced by pharmacological and non-pharmacological interventions, but these interventions are not without potential patient harm. Stratum-specific strategies are recommended for optimizing patient management to prevent VTE and bleeding events. [3]

Hospitalization during pregnancy and childbirth greatly increases the thromboembolic risk in these patients. [4] Admission to the hospital during pregnancy is associated with an 18-fold increased risk of a first VTE compared with time outside the hospital, and the risk remains high after discharge, being six-fold higher in the 28 days after discharge. [4]

The application of a protocol to assess the risk of VTE reduces the mortality and morbidity associated with these phenomena. The estimated reduction in the VTE incidence in this high-risk population would be approximately 90%, with a low incidence of adverse effects. [5] Guidelines for postpartum Thromboprophylaxis (TPX) are mostly expert-based, and the indications for TPX greatly differ between the guidelines. [6] These TPX recommendations for the postpartum period apply to patients in 0% [7] to almost 50% of cases. [5] The low number of patients studied and the retrospective nature of the published literature motivated us to perform this trial.

The goal of this study was to apply a TPX protocol based on a VTE risk score to all hospitalized pregnant women, including the postpartum period. The main objective was to prevent in-hospital deaths due to VTE and maternal death 3 months after discharge.

## Methods

This was an interventional study of hospitalized pregnant women in a single high-risk pregnancy reference center in Hospital das Clínicas, Department of Obstetrics and Gynecology, Universidade de São Paulo, São Paulo, Brazil. Patients were classified as low- or high-risk according to the Clinics Hospital VTE risk score. [8] The template for the VTE risk scoring was in an electronic formulary and was to be completed at the time of hospitalization, or the process of admission to the hospital could not be concluded.

The risk assessment was to be reapplied if the patient was hospitalized for more than 7 days. This process was followed by medical residents in most cases. The study period was from December 2014 to June 2019.

## Outcome: risk score

Various risk factors were divided into high, medium, or low risk and were assigned values of 3, 2 or 1, respectively. The final score was the sum of the values attributed to each factor present in the patient (Table 1).

**Table 1:** Risk score for Venous Thromboembolism (VTE) prevention during hospitalization in pregnancy and childbirth (Hospital das Clínicas São Paulo, Brazil, 2020).^a^

High-risk factors (Score 3)	Moderate-risk factors (Score 2)	Low-risk factors (Score 1)
Previous VTE	Previous VTE associated with a triggering factor	Dehydration
Recurrent **VTE**		Smoker (> 20 cigarettes/day)
VTE during gestation or after delivery		Multiple pregnanc
VTE linked to the use of hormones		Hyperemesis
		Age ≥ 35y and < 40y
		Parity ≥ 3 deliveries
		Any surgical procedure in the gestation or puerperium
		Gross varicose veins
***High-risk thrombophilia***	***Thrombophilia***	
Homozygous Factor V Leiden	Homocysteine > 15 ΜmoL/L	
Homozygous mutant prothrombin gene	Heterozygous Leiden factor	
Antithrombin deficiency	Heterozygous mutant prothrombin	
Thrombophilia association	Protein C deficiency	
Antiphospholipid syndrome	Protein S deficiency	
	Suspected APS	
***Cardiopathies***	***Clinical conditions***	
Mechanical valve prostheses	Cancer (in the previous 6 months)	
Atrial fibrillation or flutter	Chemotherapy (within 6 months)	
Cyanotic cardiopathies	Cyanotic pneumopathy	
Intracavitary thrombosis	Paraplegia	
Severe ventricular dysfunction	Pyelonephritis/pneumonia/puerperal infection	
Severe dilation of heart cameras	Puerperal hemorrhage >1000 mL	
	Age ≥ 40y	
	BMI ≥ 40 kg/m^2^	
	Immobility in bed more than 4 days before cesarean section	
***Other systemic diseases:***		***Risk of bleeding***
Nephrotic proteinuria (≥3.5g/24 hours prior to gestation or during the first trimester)		**Preferably using mechanical methods**
		Active bleeding
Sickle-cell anemia		Active peptic ulcers
Systemic lupus erythematosus^b^		Uncontrolled systemic arterial hypertension (> 180 × 110 mm Hg)
Acute rheumatological disease^b^		
Intestinal inflammatory disease^b^		Coagulopathy (thrombocytopenia < 70,000 or INR > 1.5)
Digestive tract cancer (pancreas and stomach); lung cancer		Allergy or heparin thrombocytopenia
		Renal insufficiency (creatinine > 1.5)
***Immobility in bed for longer than 1 week with BMI ≥ 30 kg/m***^***2***^		Cranial or ocular surgery < 2 weeks
		Cerebrospinal fluid flap < 24h
***Morbidity in previous gestation with positive thrombophilia (genetic and/or acquired)***		Hepatic/cerebral metastasis
Previous stillbirth without malformations		
Placental abruption		
Severe placental impairment:		
Zero or reverse diastole in the umbilical artery		
Restricted fetal growth (p < 3)		
Oligoamnios		

Abbreviations: APS, Antiphospholipid Syndrome; BMI, Body Mass Index.

a The high-risk group (score ≥ 3) received thromboprophylaxis with enoxaparin unless the patient had a contraindication for anticoagulation.

b Disease activity requiring hospitalization.

These risk factors were mainly adapted from the Royal College of Obstetricians and Gynaecologists (RCOG) [5] and the American College of Chest Physicians (ACCP). [9] High-risk factors for VTE in hospitalized patients were those with a risk greater than 3% (Relative Risk [RR] ≥ 6). [8] Moderate-risk factors received a score of 2 with an Odds Ratio (OR) ≥ 2 and < 6. Low-risk factors were scored 1, with an OR ≥ 1.7 and ≤ 2.

Pharmacological anticoagulation with Low-Molecular-Weight Heparin (LMWH) was indicated in patients with a VTE risk score ≥ 3. Unfractionated heparin was used when there was no availability or a contraindication to the use of enoxaparin. The high-risk group (score ≥ 3) received TPX with enoxaparin unless the patient had a contraindication for anticoagulation, such as active bleeding or high bleeding risk (Table 1). After discharge, the patient underwent TPX for 15 days. If there was a high-risk factor, TPX was prescribed for 40 days after discharge. The patients received the number of required syringes of enoxaparin when indicated, at discharge.

## Sample size calculation

The authors aimed to demonstrate that the protocol is effective in reducing the overall incidence of VTE by 50% in the high-risk group (score ≥ 3), knowing that the risk of VTE in this group is greater than 3%. [8] Using these data with a one-tailed test, a significance level of 5%, and a power of 90%, the sample size for this group (with anticoagulation) was 834 patients. When the authors increased the power of the test to 95%, the required sample was 1055 high-risk patients (www.lee.dante.br). Considering the probability of a patient loss to follow-up of 10%, the authors determined the study needed 917 patients (power, 90%) or 1160 patients (power, 95%), respectively.

## Statistical analysis

Using percentages and absolute values, the collected data were descriptively analyzed to identify the profiles of the pregnant women. One patient could have undergone more than one evaluation. Variables were compared between the low- and high-risk VTE groups, using the Chi-Square test for categorical variables. Univariate and multivariate logistic regression analyses were applied to estimate the OR and 95% Confidence Interval (95% CI) between independent covariates and VTE risk.

Interaction analysis of the main risk factors was performed using OR and Poisson regression with robust variance. All statistical analysis was performed using SPSS version 20 for Windows. A two-sided p-value of < 0.05 was considered statistically significant.

## Ethics approval

This study followed the tenets of the Declaration of Helsinki and the rules of Resolution no 196/96 of the Brazilian National Health Council. All the patients were informed of the study objectives. Only the data of those who voluntarily accepted and signed informed consent forms to participate in the study were included. This research was approved by the local ethics committee (approval: number CAAE 37431414.9.0000.0068).

## Results

**Fig. 1:**
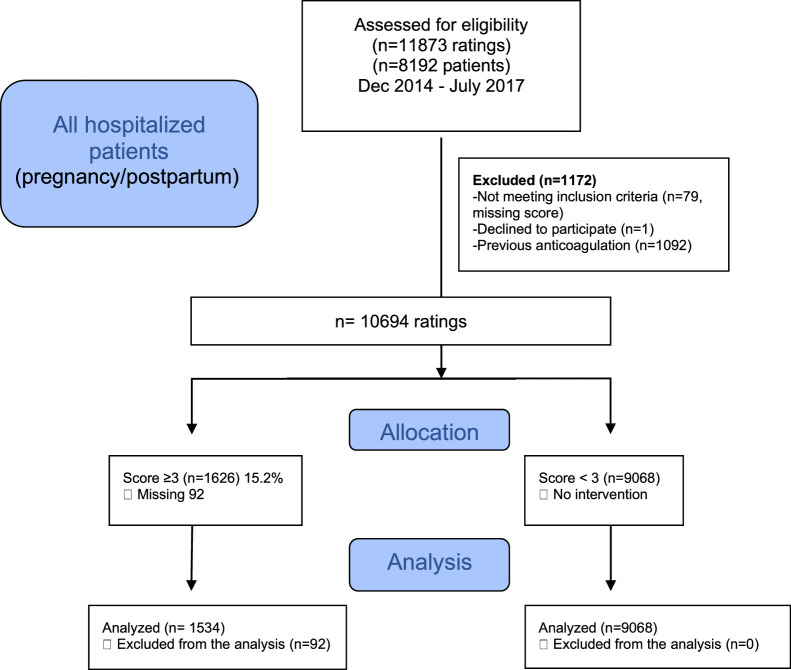
Flow diagram of all hospitalized patients (pregnancy/puerperium) assessed for eligibility.

A flowchart of the selection of patients is shown in Fig. 1. The data of 10694 cases (7212 patients) were descriptively analyzed; 1626 (15.2%) cases (in 1000 patients) were classified as high-risk (score ≥ 3) and were compared with 9608 cases (in 6212 patients) with a risk score < 3.

The main risk factors for VTE were age ≥ 35 and ≤ 39 years (OR = 1.6; 95% CI 1.4–1.8), parity ≥ 3 (OR = 3.5, 95% CI 3.0–4.0), age ≥ 40 years (OR = 4.8, 95% CI 4.1–5.6), multiple pregnancies (OR = 2.1, 95% CI 1.7–2.5), body mass index ≥ 40 kg/m^2^ (OR = 5.1, 95% CI 4.3–6.0), severe infections (OR = 4.1, 95% CI 3.3–5.1), and cancer (OR = 12.3, 95% CI 8.8–17.2) (Table 2).

**Table 2:** Main risk factors for venous thromboembolism in hospitalized pregnant women.

Risk factors	VTE score ≥ 3 (n = 1626)		VTE score < 3 (n = 9068)		Total (n = 10694)		OR (95% CI)	p
	n	%	n	%	n	%		
Age ≥35y and ≤39y	433	26.6	1663	18.3	2096	19.6	1.6 (1.4–1.8)	<0.001
Parity ≥3	419	25.8	811	8.9	1230	11.5	3.5 (3.1–4.0)	<0.001
Age ≥40y	321	19.7	439	4.8	760	7.1	4.8 (4.1–5.6)	<0.001
Multiple pregnancy	182	11.2	513	5.7	695	6.5	2.1 (1.7–2.5)	<0.001
BMI ≥40 kg/m^2^	294	18.0	375	4.1	668	6.2	5.1 (4.3–6.0)	<0.001
Surgical procedure	109	6.7	337	3.7	446	4.2	1.9 (1.5–2.3)	0.002
Severe infections	144	8.9	208	2.3	352	3.3	4.1 (3.3–5.1)	<0.001
Cancer	110	6.8	53	0.6	163	1.5	12.3 (8.8–17.2)	<0.001
Smoking >20 cigarettes	51	3.1	101	1.1	152	1.1	2.9 (2.0–4.0)	<0.001
Chemotherapy	60	3.7	14	0.2	74	0.7	24.8 (13.8–44.4)	<0.001
Sickle cell disease	67	4.1	5	0.1	72	0.7	77.9 (31.3–193.6)	<0.001
Lupus*	44	2.7	21	0.2	65	0.6	12.0 (7.1–20.2)	<0.001
Nephrotic proteinuria	49	3.0	4	0.0	53	0.5	70.4 (25.4–195.4)	<0.001
Immobility ≥7d and BMI ≥30 kg/m^2^	37	2.3	15	0.2	52	0.5	14.0 (7.7–25.7)	<0.001
Hyperemesis	8	0.5	36	0.4	44	0.4	3.4 (1.1–10.6)	0.03
Postpartum hemorrhage	26	1.6	15	0.2	41	0.4	9.8 (5.2–18.5)	<0.001
Immobility ≥4 days pre-cesarean	26	1.6	11	0.1	37	0.3	13.4 (6.6–27.1)	<0.001
Varices	9	0.6	23	0.3	32	0.03		0.045
Paraplegy	24	1.5	7	0.1	31	0.3	19.4 (8.3–45.1)	<0.001
Previous VTE (triggering factors)	22	1.4	21	0.2	43	0.4	5.9 (3.2–10.8)	<0.001
Ventriculomegaly	51	3.1	7	0.1	58	0.5	41.9 (19–92.5)	<0.001
Severe dilation of heart chambers	87	5.4	17	0.2	104	1.0	30.1 (17.8–50.7)	<0.001
Pulmonary hypertension	94	5.8	31	0.3	125	1.2	17.9 (11.9–26.9)	0.63
Cyanotic pneumopathy	1	0.1	5	0.1	6		1.1 (0.1–9.5)	0.921
Stillbirth	21	1.3	8	0.1	29	0.3	14.8 (6.5–33.5)	<0.001
Placenta abruption	5	0.3	2	0.0	7	0.1	14.0 (2.7–72.1)	0.002
Placental insufficiency	5	0.3	1	0.0	6	0.1	28.0 (3.5–239.5)	0.002
Fetal growth restriction	8	0.5	6	0.1	14	0.1	7.5 (2.6–21.6)	<0.001
Cancer (stomach)	3	0.2	1	0.0	4	0.0	16.8 (1.7–161.2)	<0.015
Hemolytic anemia	11	0.7	2	0.0	13	0.1	30.9 (6.8–139.4)	<0.001
Rheumatologic diseases (other than lupus)	10	0.6	4	0.0	14	0.1	14.0 (4.4–44.8)	<0.001
Inflammatory bowel disease	8	0.5	7	0.1	15	0.1	6.4 (2.3–17.7)	<0.001
VTE previous (postpartum)	8	0.5	0	0.0	8	0.1	41.4 (12.4–138.6)	<0.001
VTE (No triggering factors)	16	1.0	7	0.1	23	0.2	12.9 (5.3–31.3)	<0.001
VTE (with hormones)	3	0.2	0	0.0	3	0.0		<0.001
Homocysteine	1	0.1	1	0.0	2	0.0	5.6 (0.3–89.2)	0.224
Factor V Leiden heterozygous	4	0.2	3	0.0	7	0.1	7.4 (1.7–33.3)	0.009
Prothrombin heterozygous	1	0.1	3	0.0	4	0.0	1.9 (0.2–17.9)	0.591
Protein C	0	0.0	3	0.0	3	0.0		
Antiphospholipid syndrome	2	0.1	5	0.1	7	0.1	2.2 (0.4–11.5)	0.337

VTE, Venous Thromboembolism; BMI, Body Mass Index; OR, Odds Ratio.

Most cases in the high-risk group received prophylactic anticoagulation with enoxaparin (967/1534, 63%), whereas in 29.5% (453) only ambulation was prescribed. Some patients received unfractionated heparin (114, 7.4%) and were classified as ‘others’ (Table 3).

**Table 3:** Adopted therapy in patients with VTE score ≥ 3 (n = 1534).

Anticoagulation	N	%
Ambulation	453	29.5
Enoxaparin 20 mg	30	2.0
Enoxaparin 40 mg	630	41.1
Enoxaparin 60 mg	262	17.1
Enoxaparin 80 mg	45	2.9
Others	114	7.4
Total	1534	100
Missing	92	5.7

VTE, Venous Thromboembolism.

The risk factors for bleeding were reported in 299/1534 (19.5%) cases in the high-risk group and are listed in Table 4. The main risk factors were the use of antiplatelet agents (aspirin, hydroxychloroquine) (65, 4.2%), premature rupture of membranes (61, 3.7%), renal insufficiency (43, 2.8%), placenta previa (35, 2.3%), active bleeding (34, 2.2%), uncontrolled hypertension (26, 1.7%), coagulopathy (25, 1.6%), and metastasis (brain, liver) (7, 0.4%).

**Table 4:** Risk factors for major bleeding in the high-risk-for-VTE group (n =1534).

Risk factors for major bleeding	N	%
Drugs	65	4.2
PROM	61	3.7
Renal insufficiency	43	2.8
Placenta previa	35	2.3
Active bleeding	34	2.2
Hypertension	26	1.7
Coagulopathy	25	1.6
Metastasis	7	0.4
Coughing	2	0.1
Stomach ulcer	1	0.06
Total	299	19.5

VTE, Venous Thromboembolism; PROM, Premature Rupture Of Membranes.

Patients were evaluated antepartum, postpartum, or in both periods. The results are presented in Table 5. There was a significantly higher proportion of antepartum evaluations in the high-risk group (49.3%) than in the low-risk group (31.5%, p < 0.001). A total of 796 high-risk patients were scored in the postpartum period (796/1570, 50.7%). Cesarean sections were more frequent in the high-risk group (65.8%, 524/796) than in the low-risk group (56.3%, 3411/6048) (p < 0.001).

**Table 5:** Hospitalized pregnant women and VTE risk score: antepartum and postpartum evaluation.

Type of evaluation	TEV SCORE					
	< 3		≥ 3		Total	
	n	%	n	%	n	%
**Hospitalization antepartum**						
Total clinical treatment^a^	2790	78.3	774	21.7^a^	3564	34.2
**Postpartum**						
Cesarean section^b^	3411	86.6	524	13.3	3935	
Forceps	299	92.5	24	7.4	323	
Vaginal delivery	2338	90.4	248	9.5	2586	
Total postpartum	6048	88.3	796	11.6	6844	65.7
**Total**	8838	100	1570	100	10408	100

a *p* < 0.001 for total clinical treatment versus total postpartum VTE score of ≥ 3.

b Cesarean section: 65.8% (524/796) in the high-risk group versus 56.3% (3411/6048) in the low-risk group (*p* < 0.001).

Most cases of thrombosis occurred in the high-risk group (Table 5); 7/1534 (0.4%) and 3/9068 (0.03%) in the low-risk group. No patient died of VTE. Three months after discharge, 23.4% of patients could not be contacted.

## Interaction analysis of the risk factors

The interaction analysis of the risk factors is presented in Table 6. Multiple pregnancies and ages >40 years were analyzed, and there was an increase in the adjusted OR (aOR) (48.8, 95% CI 20.7–115.05). In the analysis of the interaction between multiple pregnancies and hemorrhage, there was a significant increase in aOR (35.99, 95% CI 4.33–299.14). Poisson regression with a robust estimator works with Prevalence Ratio (PR) crosscutting and binary outcomes, and the PR may be used. The same analysis for twins and hemorrhage resulted in a PR of 6 (95% CI 2.69–13.37), and the PR for twins and age > 40 years was 6.97 (95% CI 5.13–9.49).

**Table 6:** Main risk factors for venous thromboembolism in hospitalized pregnant women, interaction and prevalence ratio analysis.

Risk factors	VTE score ≥ 3 (N = 1626)		VTE score < 3 (N = 9068)		OR (95% CI)	Interaction	Prevalence ratio
						OR (95%CI)	PR (95% CI)
	N	%	N	%			
***Age ≥ 35 and ≤ 39y***	433	18.3	2096	19.6	1.6 (1.4–1.8)		
Interaction with BMI ≥ 40 kg/m^2^						20.64 (14.34–29.71)	5.94 (5.34–6.61)
Interaction with surgical procedure						4.47 (2.94–6.8)	3.03 (2.36–3.89)
Interaction with stillbirth						25.16 (2.81–225.33)	5.83 (3.75–9.07)
Interaction with severe infection						308.67 (42.54–2239.50)	7.41 (6.92–7.94)
***Age ≥ 40y***	321	4.8	760	7.1	4.8 (4.1–5.6)		
Interaction with parity ≥ 3						28.41 (20.18–40)	6.72 (6.14–7.36)
Interaction with surgical procedure						29.07 (9.76–86.53)	6.35 (5.12–7.86)
Interaction with severe infection						28.73 (8.09–101.95)	6.54 (5.05–8.48)
Interaction with multiple pregnancy						48.8 (20.7–115.05)	6.97 (5.13–9.49)
Interaction with BMI ≥ 40 kg/m^2^						28.73 (CI 8.09–101.95)	6.54 (CI 5.05–8.48)
***Multiple pregnancy***	182	5.7	695	6.5	2.1 (1.7–2.5)		
Interaction with postpartum hemorrhage						35,99 (4.33–299.14).	6 (2.69–13.37)
***Cancer***	110	0.6	163	1.5	12.3 (8.8–17.2)		
Interaction with chemotherapy						43.86 (20.91–91.96)	6.12 (5.54–6.76)

VTE, Venous Thromboembolism; PR, Prevalence Ratio; OR, Odds Ratio; CI, Confidence Interval; BMI, Body Mass Index.

The same significant increase was observed in the interaction analysis for cancer and chemotherapy (aOR = 43.86, Poisson PR = 6.12), age 35–39 years and BMI > 40 kg/m^2^ (aOR = 20.64, PR = 5.94), surgical procedure and age 35–39 years (aOR = 4.47, PR = 3.03), and surgical procedure and age > 40 years (aOR = 29.07, PR = 6.35). Other significant increases in aOR were seen in stillbirth and age 35–39 years (aOR = 25.16, PR = 5.83), severe infection and age 35–39 years (aOR = 308.67, PR = 7.41), severe infection and age > 40 years (aOR = 28.73, PR = 6.54), age > 40 years and multiparity (aOR = 28.41, PR = 6.72).

## Cases of thrombosis

Ten patients developed VTE despite risk assessment (Table 7). These patients had a high VTE risk score (7/10) in many cases, and three cases of VTE occurred in the low-risk group. Thus, the protocol failures were 7/1534 (0.4%) in the high-risk group and 3/9068 (0.03%) in the low-risk group. Patient 1 had all the complex situations of placenta previa percreta. The hemorrhagic situation was controlled on the 4^th^ day after cesarean section and hysterectomy. This was when pharmacological anticoagulation should have been initiated. This case raised the question of whether delayed or postponed anticoagulation is more appropriate after the complications of hemorrhagic shock are resolved. This patient had a high VTE risk score; however, anticoagulation was initiated only when she presented with pulmonary embolism. Patient 9 received anticoagulation after the hemorrhagic situation was resolved but was suspended enoxaparin by herself before the necessary time and had a thrombosis subsequently. Patient 4 presented with a seizure on the 25^th^ day after the cesarean section. Enoxaparin was suspended, she was readmitted to the hospital, and presented with a VTE on the 39^th^ day postpartum. Patient 5 had a high-risk score for VTE and was anticoagulated, but she had severe depression after experiencing fetal death and was predominantly on bed rest. The patient developed a pulmonary embolism and VTE. Patient 10, who died, had a thrombus in the vena cava on autopsy but had disseminated colon cancer with metastasis in the liver. Patient 7 had persistent vomiting after fetal meningomyelocele surgery. She received bed rest but experienced dehydration and cerebral venous thrombosis on the 7^th^ day after surgery. An opportunity was lost to score the patient. Special attention must be paid to twin pregnancies, as in Patient 3. A difficult extraction occurred in one malformed fetus, and the traumatic situation was probably not properly evaluated.

**Table 7:** Patients who developed thrombosis during the study period.

N	Date	Age (y)	Weight (kg)	BMI (kg/m^2^)	Risk factors	VTE score	Type of VTE	Evolution
1	25/09/2015	37	73	29	Twins, premature rupture of membranes, chorioamnionitis, bed rest, c-section, uterine atony, hysterectomy, septic shock, blood transfusion	9	PE ‒ 7^th^ day PS	Alive
2	30/06/2016	29	78	27	Fetal malformation, c-section	0	PE and DVT ‒ 7^th^ day PS	Alive
3	10/09/2016	28	73	25	Twins, difficult extraction, c-section, asthma	1	PE and DVT ‒ 11^th^ day PS	Alive
4	21/02/2017	34	60	28	Chronic renal insufficiency, seizure 25^th^ day post-normal delivery, rehospitalization	3	DVT ‒ 39^th^ PS	Alive
5	05/03/2017	33	67	31	Chronic renal insufficiency, c-section, neonatal death, depression (total bed rest), enoxaparin 100 mg	3	PE and DVT ‒ 14^th^ day PS.	Alive
6	26/06/2017	41	91	33	Increased age, gross varicose veins, suboptimal dose of heparin	3	Superficial thrombophlebitis ‒ 10^th^ day after vaginal delivery	Alive
7	22/01/2018	30	66	23	Surgery in pregnancy (correction of meningomyelocele) postoperative vomiting, bed rest, dehydration	2	Cerebral venous thrombosis ‒ 7^th^ PS (seizure)	Alive
8	18/03/2018	42	105	35	Increased age, gross varicose veins in left leg	3	Superficial thrombophlebitis ‒ 2^nd^ day post-normal delivery	Alive
9	25/09/2018	31	112	38	Emergency c-section, uterine atony, hemorrhage, multiparity, DVT in the first trimester of pregnancy, autoimmune disease, smoking, withholding of anticoagulation for 3 days after c-section	7	PE ‒ 26^th^ day PS	Alive
10	04/02/2018	34	120	38	Metastatic colon cancer, previous VTE, c-section in the 35^th^ week of pregnancy, sepsis, enoxaparin 40 mg/day	7	Vena cava Thrombosis (post-mortem diagnosis).	Dead ‒ 15^th^ day PS

BMI, Body Mass Index; c-section, cesarean section; VTE, Venous Thromboembolism, PE, Pulmonary Embolism; PS, Post-Surgery; DVT, Deep Venous Thrombosis.

## Adverse effects of thromboprophylaxis

During the study period, 4 patients (4/1648, 2.4%) presented with serious hemorrhagic complications during prophylactic anticoagulation, and 2 patients presented with large hematoma of the abdominal wall after the cesarean section. Both patient groups received enoxaparin at a dose of 40 mg/day. The first patient (paraplegic patient), after undergoing surgery, exerted notable effort while moving from the bed to the wheelchair to see her baby in the neonatal intensive care center, thus predisposing her to bleed risk. In the second patient, the identified risk factor was allergic coughing after surgery. The third patient had renal insufficiency, cesarean section, and uterine atony that required puerperal hysterectomy. On the second day postpartum, she presented with an acute hemorrhagic abdomen and underwent a laparotomy. The risk factors identified were enoxaparin 20 mg, renal insufficiency, and the use of aspirin in the postpartum period (it was not suspended after the cesarean section). The fourth patient underwent a mastectomy for breast cancer in the 5^th^ month after delivery. Enoxaparin 40 mg was administered despite the large amount of bloody drainage flowing from the surgical drain. Some hours after enoxaparin was administered, the bleeding worsened, and another surgery was necessary to achieve hemostasis. LMWH prophylaxis was not associated with an increased risk of major antepartum (0.2% with and 0.6% without LMWH; RR=0.34; 95% CI 0.04–3.21) or peripartum hemorrhage (2.5% with and 3.0% without LMWH; RR=0.82; 95% CI 0.36–1.86). 9, 10, 11, 12

## Discussion

Several risk factors have been identified for postpartum VTE; however, the level of evidence to guide TPX for postpartum or hospitalized pregnant women remains low, and there is no optimal preventive strategy. [6] Many guidelines have been issued by different medical societies, including RCOG, [5] ACCP, [9] the American College of Obstetricians and Gynecologists (ACOG), [13] and the American Society of Hematology. [7] However, recommendations for prophylaxis differ greatly between the various guidelines for pregnant and postpartum women with clinical risk factors for VTE. The guidelines of the Hospital das Clínicas, University of São Paulo, Brazil for the obstetric ward were adapted from the RCOG [5] and ACCP [9] guidelines. This was necessary due to the very high-risk population admitted to the ward both during pregnancy and in the postpartum period, with social vulnerability. There was also an urgent need for a standardized protocol since a growing number of in-hospital maternal deaths from pulmonary embolisms had occurred before the establishment of this systematic VTE risk assessment. The hospital is a tertiary center, and in recent years, the number of patients with cancer in pregnancy has increased exponentially, as has the profile of patients with obesity, increased maternal age, and multiple pregnancies. These are very special populations that are not always studied in the other guidelines. [6] The analysis of the interaction between these risk factors showed their ability to increase the risk and lethality of VTE.

The use of mechanical devices with intermittent Pneumatic Compression (IPC) in addition to standard prophylaxis for VTE prevention in very high-risk patients results in a significantly lower incidence of asymptomatic venous thrombosis. [14] However, the use of IPC alone is not sufficient to prevent VTE in high-risk patients. [15]

The strength of the present study was the high number of patients who were scored for VTE risk in a prospective study. Hospitalizations for clinical treatment were frequent and corresponded to almost half of the high-risk cases. This is not always clear in other studies, and in fact, most of the studies on VTE risk score only used the postpartum VTE risk score. 16, 17, 18

The score application indicated the use of TPX in 15.2% of all the evaluations. Considering the high-risk population studied, this is a reasonable and cost-effective result. The RCOG [5] and ACCP [9] protocols would have indicated TPX in approximately 30%–50% and 1%–2% of cases, respectively, in this same population. In-hospital maternal deaths due to pulmonary embolisms were eliminated, and protocol failure was acceptable. A constant audit of the cases led to improvement of staff awareness, mainly by discussing the cases with the medical residents and multidisciplinary staff.

The main risk factors in this study have also been confirmed in other studies. The risk due to increasing maternal age and obesity is now acknowledged worldwide. [19,20] Multiparity and multiple pregnancies are risk factors that have long been studied. [21] The magnitude of the increase in risk depends on the nature and number of risk factors. 17, 18, 19 Most clinical factors have only a modest effect on risk, with a non-notable increase in absolute risk to 0.1%. How combinations of risk factors affect VTE risk has not been well studied; for most risk factors, it is unclear whether the risks are additive or multiplicative. [6]

The high incidence of cancer during pregnancy and the postpartum period in the present study possibly occurred because the hospital is a reference center for these patients. This incidence and diversity of cancers during pregnancy have markedly increased in recent years, with breast cancer being a predominant occurrence. [22,23] This fact and the possibility of chemotherapy during pregnancy make these patients have a high risk of VTE during hospitalization for clinical treatment or delivery. Many cancer guidelines have documented this risk. [24]

Cesarean section was not considered a risk factor in this study. This is an area of much debate in VTE prophylaxis. [25] The Canadian guidelines consider only emergency cesarean section as an intermediate risk factor. [26] The authors suppose that the prospective and systematic evaluation of all pregnant patients that were hospitalized detected the main risk factors for VTE. There are some special situations where pelvic damage, trauma, or prolonged cesarean section leads to a greater VTE risk. [27] In most cesarean sections, the procedure is safe and does not increase the risk of VTE, as can be seen in this analysis.

The high-risk VTE group had an approximately 20% risk of hemorrhage in the present study. Sometimes, it is possible to perform TPX, and at other times, the very high risk of bleeding does not allow pharmacologic therapy. In this study, 41% of the patients with risk factors received TPX. Bleeding events were not significantly more often reported for enoxaparin than for untreated controls (RR=1.35, 95% CI 0.88–2.07) in one meta-analysis. [12]

The rate of protocol failure was very low, considering this high-risk group of patients. This result is very similar to that of other risk scores, not only in obstetrics. The incidence of VTE in the general obstetric population is 1–2/1000 deliveries or 0.1% or 0.2%. The mean estimated risk of VTE was 0.07% in all participants in a study of postpartum patients, 0.12% in those with recommended TPX according to the RCOG, [5] and 0.20% among women after cesarean delivery, with recommended TPX by the ACOG [13] and ACCP [9] guidelines.

The risk score in this study assumed an OR of >6 as a high risk for VTE or a VTE probability of ≥ 3% in this population. [14] Many patients had many associated risk factors, and the final risk was difficult to estimate. The 3% risk of VTE in the high-risk group was reduced to 0.4% by the intervention (anticoagulation), resulting in an 87% reduction in risk.

The number Needed To Treat (NNT) was 3.38 (1/0.3-0.00043) or 3 patients need to be treated to prevent 1 case of VTE, considering the formula NNT = 1/(Iu–Ie), where Iu is the incidence of VTE in an unexposed-to-treatment group, which was 3%, [7] and Ie is the incidence in the treated group or 7 VTE cases in 1626 evaluations. This NNT number is low if the authors consider that half of the cases in the high-risk group had a risk greater than 3%. [28,29]

One limitation of the study is that the incidence of VTE may have been higher. A systematic venous doppler ultrasound of the legs 40 days after discharge would be required to exclude asymptomatic VTE. Three months after discharge, almost one-quarter of the patients could not be contacted. The studied population was socially vulnerable, and the loss to follow-up after 3 months was probably because cell phones are not always turned on for economic reasons. Thus, it is likely that some cases of VTE may have been missed; however, the impact on maternal mortality could be assessed. There is a committee for maternal mortality in the hospital and in the city that discusses these cases, and the authors would have been communicated of any death due to VTE.

## Conclusions

This VTE risk score resulted in a 15% indication for pharmacological VTE prophylaxis and was effective in preventing maternal death from VTE. Maternal age, notable multiparity, obesity, severe infections, and cancer were the main risk factors for VTE. Protocol failure was very low in both the high- and low-risk groups. This scoring model, which does not consider cesarean section as a risk factor, seems effective. Collaboration with a multidisciplinary approach is fundamental for the safety of patients and VTE prevention. Each case of thrombosis should be fully investigated and can provide an opportunity for improvement in patient care.

## Authors’ contributions

Guarantor statement: Venina Barros. Author contributions: Venina Barros, Fernanda S Baptista, Maria Rita F L Bortolotto, and Ana Maria Kondo – project conception, implementation, development, and approval; Venina Barros, Fernanda S Baptista, Rossana PV Francisco – data analysis and manuscript writing. Rossana Pulcineli Francisco and Stela Verzinhasse – manuscript revision, sample size calculation, and statistical analysis. Venina, Fernanda Maria Rita, Ana Maria, Rossana, and Stela – collaboration regarding the final approval of the version to be published.

## Conflicts of interest

VENINA BARROS - speaker of Sanofi-Aventis, Cardinal Health and CSL-Viphor

## References

[1] Heit JA, Crusan DJ, Ashrani AA, Petterson TM, Bailey KR. (2017). Effect of a near-universal hospitalization-based prophylaxis regimen on annual number of venous thromboembolism events in the US. Blood.

[2] Nicholson M, Chan N, Bhagirath V, Ginsberg J. (2020). Prevention of venous thromboembolism in 2020 and beyond. J Clin Med.

[3] Public Health Webinar Series on Blood Disorders - 2020 | CDC, https://www.cdc.gov/ncbddd/blooddisorders/webinar-2020.html.9

[4] Abdul Sultan A, West J, Tata LJ, Fleming KM, Nelson-Piercy C, Grainge MJ (2013). Risk of first venous thromboembolism in pregnant women in hospital: population-based cohort study from England. BMJ.

[5] Royal College Obstetricians and Gynaecologists (2015).

[6] Gassmann N, Viviano M, Righini M, Fontana P, Martinez de Tejada B, Blondon M. (2021). Estimating the risk thresholds used by guidelines to recommend postpartum thromboprophylaxis. J Thromb Haemost.

[7] Bates SM, Rajasekhar A, Middeldorp S, McLintock C, Rodger MA, James AH (2018). American Society of Hematology 2018 guidelines for management of venous thromboembolism: venous thromboembolism in the context of pregnancy. Blood Adv.

[8] Clinicaltrials.gov. Thromboprophylaxis in pregnant women in hospital: a prospective clinical trial. Available from: https://clinicaltrials.gov/ct2/show/NCT02600260, accessed in 03/01/2023.

[9] Bates SM, Greer IA, Middeldorp S, Veenstra DL, Prabulos A-M, Vandvik PO (2012). VTE, Thrombophilia, Antithrombotic Therapy, and Pregnancy: Antithrombotic Therapy and Prevention of Thrombosis. Chest.

[10] Bates SM, Middeldorp S, Rodger M, James AH, Greer I (2016). Guidance for the treatment and prevention of obstetric-associated venous thromboembolism. J Thromb Thrombolysis.

[11] Cox S, Eslick R, McLintock C. (2019). Effectiveness and safety of thromboprophylaxis with enoxaparin for prevention of pregnancy-associated venous thromboembolism. J Thromb Haemost.

[12] Jacobson B, Rambiritch V, Paek D, Sayre T, Naidoo P, Shan J (2020). Safety and efficacy of enoxaparin in pregnancy: a systematic review and meta-analysis. Adv Ther.

[13] ACOG (2018). ACOG Practice Bulletin No. 196: thromboembolism in pregnancy. Obstet Gynecol.

[14] Lobastov K, Sautina E, Alencheva E, Bargandzhiya A, Schastlivtsev I, Barinov V (2021). Intermittent Pneumatic Compression in Addition to Standard Prophylaxis of Postoperative Venous Thromboembolism in Extremely High-risk Patients (IPC SUPER). Ann Surg.

[15] Nagata C, Tanabe H, Takakura S, Narui C, Saito M, Yanaihara N (2015). Randomized controlled trial of enoxaparin versus intermittent pneumatic compression for venous thromboembolism prevention in Japanese surgical patients with gynecologic malignancy. J Obstet Gynaecol Res.

[16] Burrows RF, Gan ET, Gallus AS, Wallace EM, Burrows EA. (2001). A randomized double-blind placebo-controlled trial of low molecular weight heparin as prophylaxis in preventing venous thrombotic events after caesarean section: a pilot study. BJOG.

[17] Cavazza S, Rainaldi MP, Adduci A, Palareti G. (2012). Thromboprophylaxis following cesarean delivery: one site prospective pilot study to evaluate the application of a risk score model. Thromb Res.

[18] Sultan AA, West J, Grainge MJ (2016). Development and validation of risk prediction model for venous thromboembolism in postpartum women: multinational cohort study. BMJ.

[19] Klovaite J, Benn M, Nordestgaard BG. (2015). Obesity as a causal risk factor for deep venous thrombosis: a Mendelian randomization study. J Intern Med.

[20] Virkus RA, Løkkegaard E, Lidegaard Ø, Langhoff-Roos J, Nielsen AK, Rothman KJ (2014). Risk factors for venous thromboembolism in 1.3 million pregnancies: a nationwide prospective cohort. PLoS One.

[21] Jacobsen AF, Skjeldestad FE, Sandset PM. (2008). Incidence and risk patterns of venous thromboembolism in pregnancy and puerperium-a register-based case-control study. Am J Obstet Gynecol.

[22] Hase EA, Barros VIPVL, Igai AMK, Francisco RPV, Zugaib M. (2018). Risk assessment of venous thromboembolism and thromboprophylaxis in pregnant women hospitalized with cancer: Preliminary results from a risk score. Clinics (Sao Paulo).

[23] Poggio F, Tagliamento M, Pirrone C, Soldato D, Conte B, Molinelli C (2020). Update on the management of breast cancer during pregnancy. Cancers.

[24] Walker AJ, West J, Card TR, Crooks C, Kirwan CC, Grainge MJ. (2016). When are breast cancer patients at highest risk of venous thromboembolism? a cohort study using English health care data. Blood.

[25] Dargaud Y, Rugeri L, Fleury C, Battie C, Gaucherand P, Huissoud C (2017). Personalizedthromboprophylaxis using a risk score for the management of pregnancies with high risk of thrombosis: a prospective clinical study. J Thromb Haemost.

[26] Chan WS, Rey E, Kent NE (2014). VTE in Pregnancy Guideline Working Group, Society of Obstetricians and Gynecologists of Canada. Venous thromboembolism and antithrombotic therapy in pregnancy. J Obstet Gynaecol Can.

[27] Middeldorp S, Naue C, Köhler C. (2022). Thrombophilia, thrombosis and thromboprophylaxis in pregnancy: For what and in whom?. Hamostaseologie.

[28] Bandolier. Number needed to treat. [cited 2021 Sep 7]. Available from: http://www.bandolier.org.uk/booth/glossary/NNT.html.

[29] Nuovo J, Melnikow J, Chang D. (2002). Reporting number needed to treat and absolute risk reduction in randomized controlled trials. JAMA.

